# Oxidized Perilla and Linseed Oils Induce Neuronal Apoptosis by Caspase-Dependent and -Independent Pathways

**DOI:** 10.3390/foods9050538

**Published:** 2020-04-26

**Authors:** Yuki Ueno, Yoshiyuki Kawamoto, Yamato Nakane, Risa Natsume, Kyoko Miura, Yui Okumura, Takashi Murate, Emi Hattori, Toshihiko Osawa

**Affiliations:** 1 Department of Health and Nutrition, Faculty of Psychological and Physical Science, Aichi Gakuin University, 12 Araike Iwasaki-cho, Nisshin, Aichi 470-0195, Japan; 2 Department of Biomedical Sciences, Collage of Life and Health Sciences, Chubu University, Kasugai, Aichi 487-8501, Japan

**Keywords:** alpha-linolenic acid, oxidative stress, apoptosis

## Abstract

Alpha-linolenic acid (ALA), a polyunsaturated fatty acid, is involved in bioregulatory functions. In recent years, the health-promoting effects of vegetable-derived edible oils rich in ALA have attracted attention. ALA has a variety of physiological effects such as anti-arteriosclerotic and antiallergic properties, but is prone to oxidation. Therefore, safety concerns exist with regard to adverse effects on humans induced by its oxides. However, the effects on neuronal cells induced by oxidized ALA-rich oils, such as perilla and linseed oils, have not been fully investigated. This information is very important from the viewpoint of food safety. In this study, we investigated the effects of oxidized perilla and linseed oils, which are rich in ALA, on the toxicity of neuronal SH-SY5Y cells. Perilla and linseed oils were significantly oxidized compared with other edible vegetable oils. These oxidized oils induce neuronal cell death and apoptosis via caspase-dependent and -independent pathways through reactive oxygen species (ROS) generation. Furthermore, they suppressed neurite outgrowth. These results suggest that oxidized perilla and linseed oils have the potential to cause neuronal loss and ROS-mediated apoptosis, and thus may affect the onset and progression of neurodegenerative disorders and other diseases.

## 1. Introduction

Polyunsaturated fatty acids, which are easily oxidized, promote oxidative stress in cells and tissues, and are responsible for the onset and progression of lifestyle diseases [1,2]. Peroxides and aldehydes from the peroxidation of polyunsaturated fatty acids are cytotoxic [3], increase intracellular reactive oxygen species (ROS) generation in neuronal cells [4], and cause neurodegenerative diseases [5]. Recently, linseed and perilla oils have been focused on for their ability to promote human health. Compared with other plant oils, perilla and linseed oils contain large amounts of alpha-linolenic acid (ALA), a major polyunsaturated fatty acid (50–60%) [6]. ALA is readily oxidized to generate related hydroperoxides and aldehydes, such as 9-hydroperoxide (9-OOH), 12-OOH, 13-OOH, 16-OOH [7], acrolein, and crotonaldehyde [8]. The oxidation reaction rate of plant oils differs depending on the constituent fatty acids. Oxidative stress is involved in the development of neurodegenerative diseases [9], and suppression of neuronal cell death is considered to be important for inhibiting disease progression.

ALA has multiple physiological actions, such as the reduction of serum triglycerides and blood pressure [10,11]. Furthermore, studies using cultured neuronal SH-SY5Y cells have reported that ALA has a protective effect against oxidative stress damage caused by hydrogen peroxide [12] and neurotoxicity caused by amyloid β [13]. Hence, the use of ALA-rich plant oils as functional foods has increased remarkably in recent years. However, ALA is easily oxidized by heating, and it is unclear whether ALA-rich edible plant oil that has been subjected to heat-induced oxidation causes toxicity and dysfunction of neuronal cells. The oxidation of ALA-rich oils occurs during use or storage. If oxidized oils induce injury of neuronal cells, there is concern that oxidative stress-related diseases, such as neurogenerative diseases, may result from ingesting the related oxidants. 

Neurodegenerative diseases such as Alzheimer’s disease and Parkinson’s disease are accompanied by neuronal apoptosis [14,15], for which one cause is oxidative stress [16,17]. Lipid peroxides enhance ROS production within the cell, which inhibits cell function and promotes apoptosis [18,19]. ROS-induced apoptosis occurs via a caspase-dependent pathway [20] and caspase-independent pathway induced by flavoproteins, such as apoptosis-inducing factor (AIF) [21]. Oxidized ALA-rich oil is likely to be involved in the hypofunction of neuronal cells, which is a very important concern for food safety. However, the mechanism by which oxidized ALA-rich oil induces apoptosis in neuronal cells remains unclear.

We hypothesized that ALA-rich plant oil oxidants cause neuronal cell toxicity capable of inducing the onset and progression of neurodegenerative diseases. To validate this hypothesis, we tested the effects of heat-treated ALA-rich plant oils on neuronal cells. Herein, we investigated intracellular ROS generation, cell viability, mitochondrial dysfunction, and apoptosis signaling (focusing on both caspase-dependent and -independent pathways) in the human neuroblastoma cell line SH-SY5Y.

## 2. Materials and Methods

### 2.1. Reagents

Edible plant oils (canola oil, corn oil, extra virgin olive oil, grape seed oil, linseed oil, perilla oil, rice bran oil, safflower oil, sesame oil (non-roasted), sesame oil (roasted) and soybean oil) were obtained from a local supermarket (Aichi, Japan). N-acetyl-cysteine (NAC) was obtained from Sigma-Aldrich (St. Louis, MO, USA). Caspase-3 inhibitor (Ac-DNLD-CHO), caspase-9 inhibitor (Ac-LEHD-CHO), and pan-caspase inhibitor (Z-VAD-FMK) were purchased from Peptide Institute (Osaka, Japan). Vitamin E was obtained from Fujifilm Wako Pure Chemical Corporation (Osaka, Japan). Anti-AIF antibody was purchased from Proteintech (Rosemont, IL, USA). Anti-Bax, anti-Bcl-2, anti-caspase-3, anti-COX IV, anti-cytochrome c, and anti-poly (ADP-ribose) polymerase (PARP) antibodies, as well as a secondary horseradish peroxidase-conjugated anti-rabbit antibody, were obtained from Cell Signaling Technology (Danvers, MA, USA).

### 2.2. Oxidation of Edible Plant Oil and Oxidative State Assessment

Each edible plant oil was heated to 60 °C for 0–10 d. The oxidative state of oils was evaluated by gravimetric, thiobarbituric acid (TBA), and peroxide value (POV) methods. Oils used for gravimetiric methods were heated for 0, 2, 4, 6, 8, and 10 d; for the POV method, oils were heated for 0, 1, 2, and 3 d; for other experiments, oils were heated for 0 and 3 d. Gravimetric analysis assessed the weight change in plant oils. To perform the TBA method, 1 g of oil, 1 mL of trichloroacetic acid (TCA) solution (20% TCA in 2 M phosphoric acid), and 2 mL of 0.01 M TBA solution were heated in boiling water for 30 min. After cooling, 2 mL of isopropanol and 1 mL of pyridine were added and mixed. Next, the sample was centrifuged for 15 min at 1500× *g*. The upper layer was collected and its absorbance at 532 nm was measured. POV values of oils were quantified by the iodometric titration method. Briefly, an aliquot of oil was accurately weighed (0.5 to 5 g) into a flask and 12.5 mL of chloroform-acetic acid (2:3, v/v) solution were added. After substituting the air in the flask with N_2_ gas, 0.5 mL of saturated potassium iodide aqueous solution was added and mixed for 1 min. Following incubation in the dark for 10 min, 15 mL of ultrapure water were added and mixed vigorously. Next, 0.5 mL of 1% starch indicator was added, and the mixture was titrated with 0.01 M sodium thiosulfate solution until the initial brown color of the solution turned pale yellow. The POV was calculated using the following equation, a: Titration volume (mL); f: Factor (1.001); S: Sample size (g)

POV (meq/kg) = (a × f × 10)/S,(1)

### 2.3. Cell Line and Culture

The human neuroblastoma cell line SH-SY5Y was purchased from American Type Culture Collection (Manassas, VA) and maintained in Dulbecco’s Modified Eagle’s Medium (DMEM; Wako) containing 10% heat-inactivated fetal bovine serum, 100 units/mL penicillin, and 100 μg/mL streptomycin. Cell cultures were incubated at 37 °C and 5% CO_2_ in a humidified atmosphere. Cells from passages 3 to 10 (counted from their arrival) were used for the current experiments.

### 2.4. Cell Viability Assay

SH-SY5Y cells (2 × 10^4^ cells/well) were transferred into a 96-well plate and cultured for 24 h. To assess the effect of antioxidants, cells were pretreated with NAC (10 mM) or vitamin E (10 μM) for 12 h or 2 h, respectively, before subsequent oil treatment. After the administration of heat-treated or untreated edible plant oil in DMEM containing 1% fatty acid-free bovine serum albumin (BSA), cells were incubated for 24 h. Cells were washed three times with PBS, and viable cells were examined with a WST-8 assay using a Cell Counting Kit-8 (Dojindo Laboratories, Kumamoto, Japan) according to the manufacturer’s protocol.

### 2.5. Analysis of Intracellular ROS

SH-SY5Y cells (5 × 10^5^ cells/well) were exposed to heat-treated perilla oil, linseed oil, or sesame oil (60 °C, 3 d, 1 mg/mL) in DMEM containing 1% fatty acid-free BSA. After 3 h, cells were treated with 10 µM chloromethyl derivative of 2′,7′-dichlorodihydro fluorescein diacetate (CM-H_2_DCFDA) for 45 min at 37°C. Cells were then washed and resuspended in phosphate-buffered saline (PBS) containing 1% BSA. ROS content in these cells was examined by flow cytometry with a FACS Canto (Becton Dickinson, Franklin Lakes, NJ, USA; Ex 495 nm/Em 535 nm). Data were analyzed by FlowJo Software (ver.7.6.5, TreeStar, Ashland, OR, USA). 

### 2.6. Quantification of Neurite Formation and Cell Number

To promote neurite formation, SH-SY5Y cells were cultured with all-trans retinoic acid (10 μM) for 4 d. To assess the effect of antioxidants, cells were pretreated with NAC (10 mM) for 14 h before subsequent administration of heat-treated oils for 24 h. Images were acquired randomly from at least three fields in each well with an inverted microscope. The resulting image data were exported to a computer for measurement of neurite lengths with ImageJ software (ver. 1.51j8; NIH, Bethesda, MD, USA). Cells with neurites longer than 50 μm were counted as neurite-positive. At least 150 cells were counted per sample and mean ± SD was calculated. 

### 2.7. Mitochondrial Membrane Potential Analysis by Fluorescence Microscopy

Mitochondrial membrane potential was monitored by JC-1 dye (Cayman Chemicals, Ann Arbor, MI, USA) according to the manufacturer’s instructions. JC-1 dye exhibits an aggregated form that accumulates in mitochondria in response to changes in mitochondrial membrane potential (ΔΨm). At high ΔΨm, the JC-1 concentration increases and exhibits high aggregation (J-aggregated), which accumulates in mitochondria and emits red fluorescence (excitation/emission = 540/570 nm), whereas, at a low concentration (resulting from low ΔΨm), JC-1 exists in a monomeric form (J-monomer) in the cytoplasm, emitting a low green fluorescence intensity (excitation/emission = 485/535 nm). Therefore, J-monomers are detected in the cytosol of apoptotic cells, while J-aggregates exist in the mitochondria of non-apoptotic cells. Fluorescence images were obtained using a fluorescence microscope (DP73, Olympus, Tokyo, Japan). 

### 2.8. Detection of Apoptotic Cells

Cells were fixed with 70% ethanol, incubated with 2 mg/mL RNase A for 30 min at 37 °C, and stained with 20 µg/mL propidium iodide (PI) for 3 min at room temperature in the dark. Apoptotic cells were detected by flow cytometry with a FACS Canto. Cells exhibiting a sub-G1 peak, i.e., whose fluorescence intensity was lower than G1 peak due to the existence of fragmented DNA, were categorized as apoptotic. Percentages of sub-G1 peak cells were analyzed by FlowJo Software.

### 2.9. Apoptosis Analysis by Flow Cytometry

Apoptotic cells were quantified using an Annexin V-FITC Apoptosis Detection Kit (Nacalai Tesque, Kyoto, Japan) in accordance with the manufacturer’s instructions. Cells were examined by flow cytometry with a FACS Canto. During the early stages of apoptosis, phosphatidylserine translocates from the intracellular plasma membrane to the cell surface, whereby it can specifically bind FITC-labeled Annexin V. During the later stages of apoptosis, PI can readily move across the cell membrane and bind to cellular DNA. Therefore, when cells are double stained with Annexin V-FITC and PI, four different cell populations may be observed: (i) viable cells stained with neither Annexin V-FITC nor PI, (ii) early apoptotic cells stained with Annexin V-FITC only, (iii) late apoptotic cells stained with both reagents, and (iv) necrotic cells stained with PI only. An illustration of the expected staining of live and dead cell populations is shown in Figure S1 (upper panel). 

### 2.10. Subcellular Fractionation

To separate mitochondrial and nuclear fractions, subcellular fractionation was performed using a kit (BioVision, Milpitas, CA, USA) in accordance with the manufacturer’s instructions with minor modifications. Briefly, cells were washed with ice-cold PBS and resuspended with Cytosol Extraction Buffer Mix containing dithiothreitol (1 mM) and protease inhibitor cocktail. After incubation at 4 °C for 10 min, cells were homogenized with a Dounce homogenizer on ice. The homogenate was collected and centrifuged at 700× *g* for 10 min at 4 °C. The supernatant was collected and subjected to mitochondrial fractionation. The pellet was used as the nuclear fraction. The supernatant was centrifuged at 10,000× *g* for 30 min at 4 °C. The pellet was collected (mitochondrial fraction) and resuspended in 10 µL of the Mitochondrial Extraction Buffer Mix containing dithiothreitol (1 mM) and protease inhibitors. 

### 2.11. Western Blotting

Western blotting was performed as previously described [22] with slight modifications using specific antibodies. Briefly, lysates of SH-SY5Y cells were separated by SDS-PAGE using a SuperSep Ace 5–20% gel (Wako), and the resulting proteins were transferred to a polyvinylidene difluoride membrane (Merck Millipore, Darmstadt, Germany). The membrane was blocked with 5% nonfat milk for 1 h at room temperature and then reacted with primary antibodies (all antibodies used at 1:1000 dilution) for 18 h at 4 °C, followed by reaction with the corresponding secondary horseradish peroxidase-conjugated antibody (all antibodies used at 1:1000 dilution) for 1 h at room temperature. Signals were detected by Western Lightning Plus-ECL (PerkinElmer, MA, USA). Chemiluminescence was captured using a cooled CCD Light-Capture camera system and analyzed using CS Analyzer software version 2.0 (ATTO, Tokyo, Japan).

The caspase pathway was analyzed by detecting changes in proteins cleaved upon activation (caspase-3, PARP, and AIF), translocation of cytochrome c out of mitochondria, and regulators that promote (Bax) or suppress (Bcl-2) apoptosis by western blotting.

### 2.12. Statistical Analysis

All experiments were performed in triplicate at least two independent times and the values shown represent mean ± standard deviation. Statistical analyses were performed with Statcel 3 software (OMS Publisher, Tokorozawa, Japan). Statistical differences were analyzed by Student’s *t* test for two-group comparisons, while one-way ANOVA with Dunnett’s test or Tukey–Kramer’s test was used for multiple-group comparisons. Statistical significance was defined as *p* < 0.05 or *p* < 0.01.

## 3. Results

### 3.1. Heat-treated Perilla and Linseed Oils Rapidly Reach Higher Oxidation States

**Figure 1:**
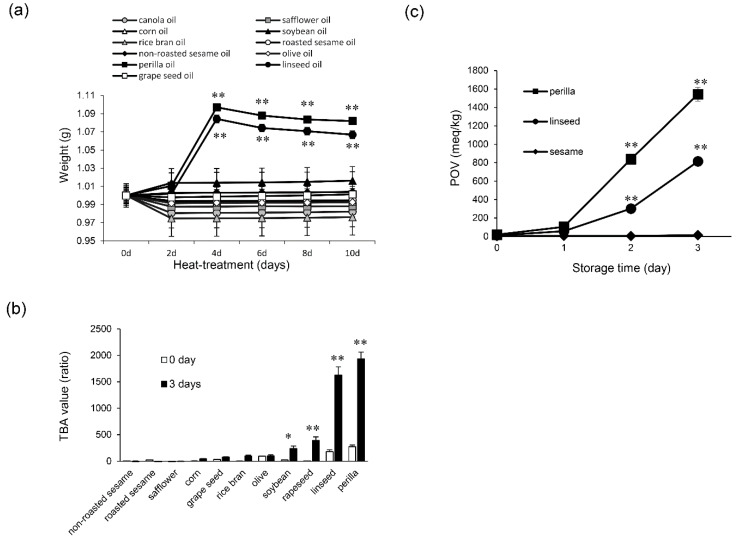
Oxidation state of edible plant oils. (**a**) Non-oxidized oil (0 d) and oxidized oil (2, 4, 6, 8 and 10 d) were evaluated for oxidation state by a gravimetric method. One-way ANOVA with Dunnett’s test was used. ** *p* < 0.01 compared with perilla oil (0 d) and linseed oil (0 d), respectively. (**b**) Non-oxidized oil (0 d) and oxidized oil (3 d) were evaluated for oxidation state by the TBA method. Student’s *t* test was used. * *p* < 0.05 and ** *p* < 0.01 compared with control (0 d). (**c**) Peroxide value (POV) of perilla, linseed, and non-roasted sesame oils during the conservation period (in days) at 60 °C. Results are expressed as mean ± SD (n = 3). One-way ANOVA with Dunnett’s test was used. ** *p* < 0.01 compared with control (0 d).

ALA-rich plant oils are rapidly oxidized, as they contain active methylene groups. To confirm the oxidative state of plant oils after heating, we performed gravimetric, TBA, and POV analyses. The results of gravimetric analysis demonstrated that the oxidative state of perilla and linseed oils, which are extremely rich in ALA, was significantly increased (** *p* < 0.01) after 4 d of heat treatment compared with unheated oil (Figure 1a). Next, the oxidative state of perilla and linseed oils heated for 3 d was assessed by the TBA method because the oxidation reaction as indicated by the gravimetric method shown in Figure 1a increased significantly after 4 d of the heat treatment. These oils showed drastically increased TBA values compared with other oils (Figure 1b). According to these results, we chose perilla and linseed oils to perform POV analysis (lipid peroxide assays). Time-course experiments employing the POV method, in which perilla and linseed oils were heated for 0 to 3 d, demonstrated significantly increased values after 2 d of heating (Figure 1c). Sesame oil (non-roasted), which contains only a low amount (< 1 %) of ALA [23], showed non-oxidative state scores in these analyses (Figure 1a–c). Based on these results, sesame oil (non-roasted) was used as a representative control of oxidation-resistant oil for further experiments.

### 3.2. Heat-treated Perilla and Linseed Oils Decreased Cell Viability and Neurite Outgrowth in SH-SY5Y Cells through ROS Generation 

**Figure 2:**
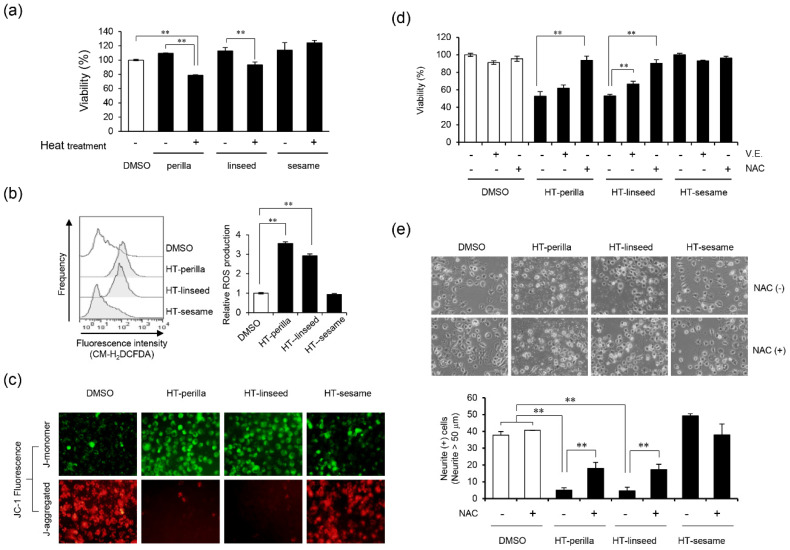
Injury of SH-SY5Y cells by heat-treated oil and the preventive effect of antioxidants. (**a**) Analysis of cell viability after exposure to heat-treated or unheated oils. After incubation with unheated (0 d) or heat-treated oil (3 d, 1 mg/mL) for 24 h, cell viability was measured using a Cell Counting Kit-8. One-way ANOVA with Tukey–Kramer’s test was used. ** *p* < 0.01 compared with DMSO treatment. (**b**) Intracellular ROS production induced by oxidized oils. SH-SY5Y cells were exposed to 1 mg/mL oxidized perilla, linseed, or sesame oil for 3 h in DMEM media containing 1% fatty acid-free BSA. Cells were stained with CM-H_2_DCFDA, and then subjected to flow cytometry analysis. The left panel shows a histogram, while the right panel shows relative ROS production levels calculated by the mean value of the fluorescence level. One-way ANOVA with Dunnett’s test was used. ** *p* < 0.01 compared with control (DMSO). (**c**) Fluorescence imaging of ΔΨm in SH-SY5Y cells by fluorescence microscopy. Cells were exposed to heat-treated oils as indicated for 24 h, and then stained with JC-1. The J-aggregated form (red, healthy) and J-monomer form (green, apoptotic) were measured at 570 and 535 nm, respectively. Representative data of at least three experiments are shown. (**d**) Cells were pretreated with NAC (10 mM, 12 h) or vitamin E (10 μM, 2 h), and then exposed to heat-treated oil (3 d, 1 mg/mL) for 24 h. Cell viability was measured using a Cell Counting Kit-8. One-way ANOVA with Dunnett’s test was used. ** *p* < 0.01 compared with control (HT-perilla or HT-linseed or HT-sesame sample group treated without NAC or vitamin E). (**e**) Neurite formation is illustrated and the percentage of neurite-positive cells is shown. SH-SY5Y cells were cultured with all-trans retinoic acid (10 μM, 4 d), then pretreated with or without NAC (10 mM, 14 h), followed by incubation with a heat-treated oil (0.5 mg/mL, 24 h). HT, “heat-treated”. One-way ANOVA with Tukey–Kramer’s test was used. ** *p* < 0.01 compared with DMSO treatment. Results are expressed as mean ± SD (n = 3).

Next, we examined whether heat-treated perilla and linseed oils could affect neuronal cell viability. After SH-SY5Y cells were incubated with heat-treated or unheated perilla, linseed, or sesame oil for 24 h, cell viability was measured. Heat-treated perilla and linseed oils significantly reduced cell viability compared with unheated perilla or linseed oil (Figure 2a, ** *p* < 0.01). Conversely, heat-treated or unheated sesame oil had no effect on cell viability. 

Intracellular ROS generation plays an important role in nervous system diseases [5]. Therefore, we examined the effects of heat-treated perilla and linseed oils on ROS generation in SH-SY5Y cells by using a ROS-sensitive fluorescent probe and flow cytometry. Heat-treated perilla and linseed oils significantly increased intracellular ROS levels compared with controls (** *p* < 0.01), whereas heat-treated sesame oil had no effect (Figure 2b). In addition, we measured mitochondrial membrane potential and damage using JC-1 fluorescence. Heat-treated perilla and linseed oils decreased the J-aggregate level and increased the J-monomer level, while heat-treated sesame oil had no effect (Figure 2c). These data suggest that heat-treated perilla and linseed oils, but not sesame oil, cause ROS accumulation and impair mitochondrial function. Next, we investigated the effect of antioxidants on neuronal cell death induced by heat-treated perilla or linseed oils. The antioxidants vitamin E and NAC significantly ameliorated the decrease in cell viability induced by heat-treated perilla and linseed oils (Figure 2d; ** *p* < 0.01). To further examine the effect of heat-treated perilla and linseed oils on neurite outgrowth, we counted neurite-positive cells after treatment. Heat-treated perilla and linseed oils, but not sesame oil, suppressed neurite outgrowth; however, NAC significantly reversed this effect (Figure 2e, ** *p* < 0.01). Throughout the experiments in this section, neurite outgrowth was unaffected by heat-treated sesame oil. These results suggest that heat-treated perilla and linseed oils inhibited neurite outgrowth by increasing intracellular ROS.

### 3.3. Heat-Treated Perilla and Linseed Oils Induced Apoptosis in SH-SY5Y Cells 

**Figure 3:**
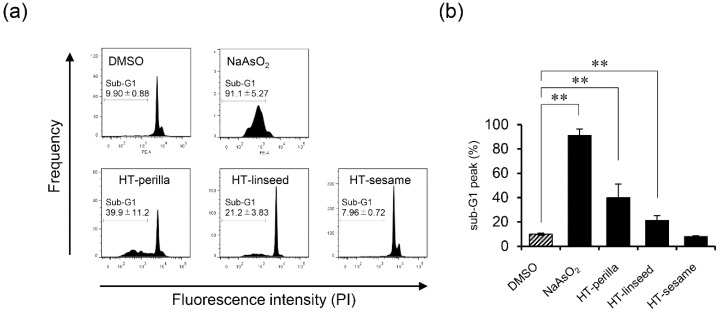
Heat-treated oils induced DNA fragmentation in SH-SY5Y cells. SH-SY5Y cells were incubated with heat-treated oils (3 d, 1 mg/mL) for 24 h. (**a**) After exposure to heat-treated oils, cells were fixed and stained with PI, and then the sub-G1 peak was examined by flow cytometry. NaAsO_2_ (1 mM) was used as an apoptosis-inducing positive control reagent. Representative histograms from at least three experiments are shown. (**b**) Percentage of sub-G1 peak. One-way ANOVA with Dunnett’s test was used. ** *p* < 0.01 compared with control (DMSO). Results are expressed as mean ± SD (n = 3).

**Figure 4:**
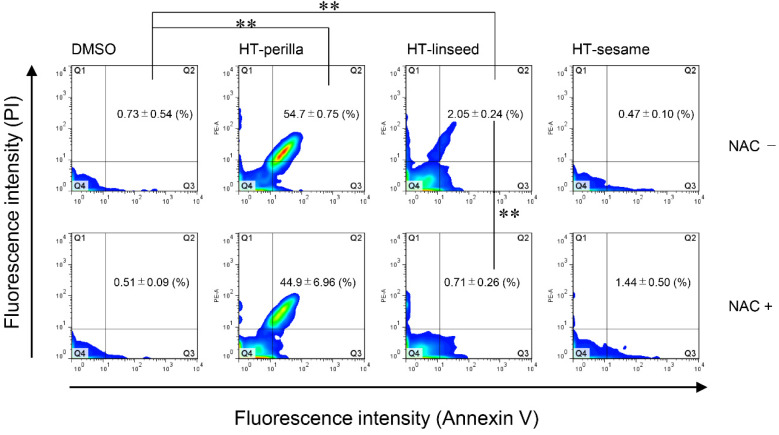
Heat-treated oils induced apoptosis in SH-SY5Y cells. SH-SY5Y cells were pretreated with or without NAC (10 mM, 12 h), and then exposed to heat-treated oils (3 d, 1 mg/mL) for 24 h. After exposure to heat-treated oils, cells were stained with Annexin V-FITC and PI, and then analyzed by flow cytometry. One-way ANOVA with Tukey–Kramer’s test was used. ** *p* < 0.01 compared with DMSO treatment. Values represent mean ± SD (n = 3).

We analyzed the sub-G1 population, which indicates cells with less DNA content as a marker of dying or dead cells, of SH-SY5Y cells incubated with heat-treated ALA-rich oils to examine DNA fragmentation induced by apoptosis using flow cytometry. Sodium arsenite (NaAsO_2_) was used as an apoptosis-inducing positive control. Heat-treated perilla and linseed oils significantly increased ** *p* < 0.01) the sub-G1 population, however, heat-treated sesame oil elicited no change (Figure 3a, b). An increase in the proportion of cells in the sub-G1 region may occur not only because of apoptosis, but also necrosis. Therefore, to confirm the induction of apoptosis, we performed an annexin V and PI double-staining assay by flow cytometry. Annexin V and PI staining analysis showed that the number of annexin V and PI double-positive cells (Q2 area) was significantly increased (** *p* < 0.01) in cultures incubated with heat-treated perilla or linseed oils. However, the increase in Annexin V and PI double-positive cells induced by heat-treated perilla oil tended to be decreased by pretreatment with the antioxidant NAC. For heat-treated linseed oil, pretreatment with the antioxidant NAC significantly abrogated (** *p* < 0.01) the increase in Annexin V and PI double-positive cells (Figure 4 and Figure S1). These results indicated that heat-treated perilla and linseed oils, but not sesame oil, induced oxidative stress-mediated apoptosis in SH-SY5Y nerve cells.

### 3.4. Effect of Heat-Treated Perilla and Linseed Oils on Caspase, PARP, and AIF

**Figure 5:**
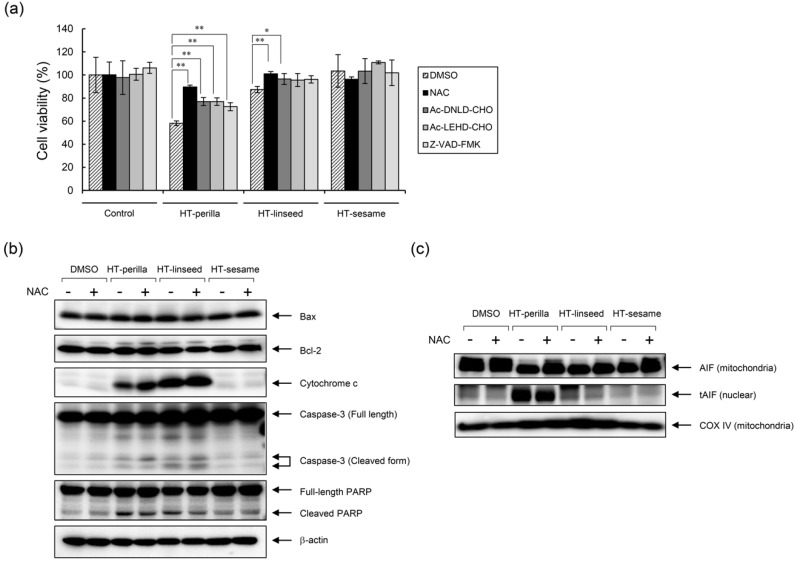
Analysis of the apoptotic mechanism induced by heat-treated oils. (**a**) The anti-apoptotic effect of caspase inhibitors and an antioxidant. SH-SY5Y cells were pretreated with a caspase-3 inhibitor (Ac-DNLD-CHO, 100 μM), caspase-9 inhibitor (Ac-LEHD-CHO, 100 μM), pan-caspase inhibitor (Z-VAD-FMK, 20 μM), or NAC (10 mM) for 12 h, and then exposed to heat-treated oils for 24 h. Cell viability was measured using a Cell Counting Kit-8. One-way ANOVA with Dunnett’s test was used. * *p* < 0.05 and ** *p* < 0.01 compared with control (DMSO). Results are expressed as mean ± SD (n = 3). (**b**,**c**) SH-SY5Y cells were pretreated with NAC (10 mM) for 12 h, and then incubated with heat-treated oils (1 mg/mL) for 24 h. (**b**) Western blot analysis was performed using the corresponding specific antibodies. Representative results of at least three experiments are shown. (**c**) Mitochondria and nuclear fractions were separated by subcellular fractionation. Western blotting was performed on mitochondrial and nuclear fractions using anti-AIF, which recognizes both truncated (tAIF) and untruncated forms of AIF. COX IV was used as loading control for the mitochondrial fraction.

To further examine whether apoptosis induced by heat-treated perilla or linseed oils was caspase-dependent, SH-SY5Y cells were incubated with the caspase-3 inhibitor Ac-DNLD-CHO, caspase-9 inhibitor Ac-LEND-CHO, or pan-caspase inhibitor Z-VAD-FMK. Observed decreases in cell viability after exposure to heat-treated perilla or linseed oils was partially ameliorated by pretreatment with each caspase inhibitor (Figure 5a). Next, we investigated the expression of apoptosis-related molecules in SH-SY5Y cells by western blotting to examine the apoptosis pathway and potential correlations to oxidative stress (Figure 5b). Bax and Bcl-2 protein levels were unaffected by heat-treated or unheated oils, whereas cytochrome c release and the cleaved form of caspase-3 were increased by heat-treated perilla and linseed oils. However, the antioxidant NAC did not affect these levels. In contrast, the cleaved form of PARP was induced by heat-treated perilla and linseed oils, but was inhibited by NAC. These results indicate that activation of cytochrome c and caspase-3 dependent signaling pathways by heat-treated perilla and linseed oils occurred in a ROS-independent manner, and the cleavage of PARP by these oils is at least partly exerted by a ROS-mediated mechanism. To further examine the mechanisms by which heat-treated perilla and linseed oils induce apoptosis, we also investigated the caspase-independent pathway and major role of AIF. Heat-treated perilla and linseed oils decreased AIF in mitochondria and increased AIF in nuclei (Figure 5c). However, nuclear levels of truncated AIF (tAIF) were decreased by NAC treatment. These results demonstrate that heat-treated perilla and linseed oils activate the AIF-mediated pathway in a ROS-dependent manner. Collectively, these results suggest that apoptosis induced in SH-SY5Y cells by heat-treated perilla or linseed oils involved both caspase-dependent and -independent pathways.

## 4. Discussion

Lipid peroxides exhibit toxicity to nerve cells [24], but only a few studies have examined the cytotoxicity of edible oils oxidized by heat treatment. In this study, we hypothesized that the oxidation of ALA-rich edible plant oils is toxic to nerve cells and induces cell death via intracellular oxidative stress. Herein, we demonstrated that plant oils rich in polyunsaturated fatty acids such as ALA, in particular perilla and linseed oils, oxidized rapidly when heated and subsequently induced apoptosis in neuronal cells.

Heating resulted in significantly more oxidation of perilla and linseed oils compared with other edible oils, as confirmed by three different methods (Figure 1a–c). Cell viability assay results demonstrated that oxidized perilla and linseed oils were toxic to nerve cells (Figure 2a). However, sesame oil did not show any toxicity at similar concentrations because it was not oxidized by heating. We also confirmed that oxidized linseed and perilla oils increased ROS production in nerve cells (Figure 2b). Moreover, these oxidized oils induced mitochondrial damage (Figure 2c), indicating that mitochondrial damage mediated the observed increase in ROS generation. As expected, the antioxidants NAC and vitamin E significantly ameliorated the decreased cell viability (Figure 2d) and inhibition of neurite extension induced by oxidized oils (Figure 2e). These results raise the possibility that heat-treated perilla and linseed oils may cause neurocytotoxicity, which is elicited by lipid peroxides.

Previous reports suggest that lipid peroxides react with proteins and nucleic acids, causing various diseases (such as neurodegenerative diseases) and reduced cell function [25]. However, the involvement of oxidized edible oils in the functional deterioration of nerve cells was unknown. In this study, we demonstrated that oxidized edible linseed and perilla oils induced apoptosis in nerve cells (Figure 3 and Figure 4). In contrast, heat-treated sesame oil did not induce apoptosis. These results indicate that ALA-derived peroxides and aldehydes in heat-treated perilla and linseed oils induce apoptosis by increasing ROS production in nerve cells. The finding that heat-treated sesame oil was not toxic is reasonable considering that antioxidants in sesame oil, such as sesaminol and α-tocopherol, can restrain peroxide generation [26,27,28]. 

Cell death induced by oxidized ALA-rich oils was partly suppressed by NAC and caspase inhibitors (Figure 5a). Cell signaling analysis focused on apoptosis-related molecules revealed that oxidized oil-induced apoptosis was not mediated by a Bcl-2- or Bax-related mechanism, but by a cytochrome c- and caspase-3-related mechanism (Figure 5b). Unexpectedly, NAC did not inhibit cytochrome c release or caspase-3 cleavage, but ameliorated PARP cleavage. These results suggest that oxidized-oil-induced apoptosis was elicited by both caspase-dependent and -independent pathways. To further analyze caspase-independent apoptosis, we examined the involvement of AIF. tAIF is directly responsible for apoptosis induction [21]. As shown in Figure 5c, oxidized oils affected AIF truncation and promoted translocation of AIF from the mitochondria to the nucleus, whereas NAC treatment partly inhibited AIF activation. These results indicate that oxidized oil-induced apoptosis was mediated by both caspase-dependent and AIF-dependent (i.e., caspase-independent) pathways. 

There are several limitations to consider in this study. Although neuronal SH-SY5Y cells retain some properties of normal cells, normal primary neuronal cells should also be examined using the same experiments. In addition, to assess the pathological or physiological effects of heat-treated oils, it will be valuable to investigate the neurotoxicity of oxidized oil by analyzing brain dysfunction, motor dysfunction, and/or reflex abnormality of lower extremity extension in animal models. As for the mechanism by which apoptosis is induced by heat-treated oils, a detailed analysis of caspase-dependent and -independent pathways induced by oxidized oils should be further performed. For instance, the endonuclease-G-dependent pathway may be an involved caspase-independent mechanism of apoptosis [29]. Furthermore, other types of cells and tissues may also be targets of oxidized edible oils. Thus, their effects should be considered. Finally, although the major unsaturated fatty acid in perilla and linseed oils is certainly ALA, we did not use ALA alone as a control. There may be a synergistic effect of oxidized ALA with other minor by-products, which determines the final outcome. Therefore, to elucidate the responsible molecule(s) contained in heat-treated perilla and linseed oils, as well as the molecular mechanism underlying nerve cell death induction, purified ALA should be used as a control and investigated in detail. Although a direct relationship between oxidized ALA-rich oil and neurodegenerative disease is not evidenced, at least an indirect relationship is predicted because acrolein (a highly reactive, α,β-unsaturated aldehyde) and its adducts were found in the brains of patients with neurodegenerative diseases [30,31,32], and ALA is the main source of acrolein formed during heating of vegetable oils [33].

In conclusion, among edible oils, those containing abundant ALA were highly susceptible to oxidation, and oxidized perilla and linseed oils exhibited apoptotic cytotoxicity in the neuronal cell line SH-SY5Y. Because these oxidized oils induced apoptosis through enhanced ROS production in nerve cells, they may participate in the onset and progression of neurodegenerative diseases. Therefore, ALA-rich edible oils should be used without heating and care should be taken to avoid oxidation.

## Supplementary Materials

The following are available online at https://www.mdpi.com/2304-8158/9/5/538/s1, Figure S1: Additional information and data of flow cytometric analysis of apoptosis using Annexin V-FITC and PI.
