# Supplementary material for: Oxidized Perilla and Linseed Oils Induce Neuronal Apoptosis by Caspase-Dependent and -Independent Pathways
Source: Foods. 2020 Apr 26;9(5):538. doi: 10.3390/foods9050538 (PMC7278870; doi:10.3390/foods9050538)
Supplement: Supplementary file 1 [file foods-09-00538-s001.pdf]

Figure S1

(a)

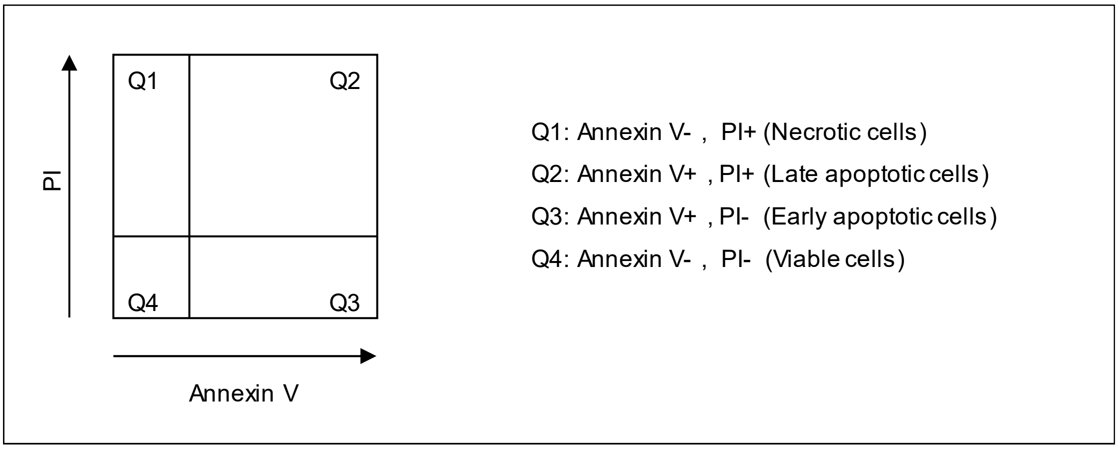

(b)

	DMSO	NAC	HT-Perilla	NAC+ HT-Perilla	HT-Linseed	NAC+ HT-Linseed	HT-Sesame	NAC+ HT-Sesame
Q1:	0.06 ± 0.05	0.06 ± 0.04	2.05 ± 0.48	6.94 ± 1.96	3.21 ± 0.75	4.29 ± 1.85	0.09 ± 0.03	0.30 ± 0.05
Q2:	0.73 ± 0.54	0.51 ± 0.09	54.7 ± 0.75	44.9 ± 6.96	2.05 ± 0.24	0.71 ± 0.26	0.47 ± 0.10	1.44 ± 0.50
Q3:	2.66 ± 1.04	2.88 ± 0.55	10.8 ± 0.97	3.39 ± 0.70	5.09 ± 0.67	7.94 ± 0.53	3.64 ± 0.58	3.67 ± 0.62
Q4:	96.6 ± 1.54	96.6 ± 0.61	32.4 ± 1.15	44.9 ± 8.39	89.6 ± 1.34	87.1 ± 2.63	95.8 ± 0.50	94.6 ± 1.04

Figure S1. Upper panel (a) shows the state of each cell in the area. Lower list (b) shows percentages of viable (Q4), early apoptotic (Q3), late apoptotic (Q2), and necrotic (Q1) cells under the indicated treatment. Values represent mean ± SD (n = 3).
